# Formaldehyde vapour fixation enables multiscale phase-contrast imaging and histological validation of human-sized lungs

**DOI:** 10.1038/s41598-025-23903-1

**Published:** 2025-10-20

**Authors:** Christian Dullin, Johanna Reiser, Willi L. Wagner, Elena Longo, Marko Prašek, Adriano Contillo, Nicola Sodini, Diego Dreossi, Paola Confalonieri, Francesco Salton, Marco Confalonieri, Elisa Baratella, Maria Assunta Cova, Claudia V. Benke, Md Motiur Rahman Sagar, Lorenzo D’Amico, Jonas Albers, Angelika Svetlove, Elizabeth Duke, Tatiana Flisikowska, Krzysztof Flisikowski, Mark O. Wielpütz, Jürgen Biederer, Hans-Ulrich Kauczor, Frauke Alves, Fabrizio Zanconati, Giuliana Tromba

**Affiliations:** 1 Department of Clinical and Interventional Radiology, University Medical Center Goettingen, Goettingen, Germany; 2 Translational Molecular Imaging, Max-Planck-Institute for Multidisciplinary Sciences, Goettingen, Germany; 3 Elettra-Sincrotrone Trieste S.C.p.A., Basovizza, Italy; 4 Department of Diagnostic and Interventional Radiology, University Hospital Heidelberg, Heidelberg, Germany; 5 Translational Lung Research Center Heidelberg (TLRC-H), German Center for Lung Research (DZL), Heidelberg, Germany; 6 Diagnostic Radiology and Neuroradiology, University Medicine Greifswald, Greifswald, Germany; 7 Department of Pulmonology, University Hospital of Cattinara, Trieste, Italy; 8 Department of Medical, Surgical and Health Sciences, University Hospital of Cattinara, Trieste, Italy; 9 Department of Radiology, University Hospital of Cattinara, Trieste, Italy; 10 European Molecular Biology Laboratory (EMBL), Hamburg Unit c/oDESY, Hamburg, Germany; 11 Department of Molecular Life Sciences, Chair of Livestock Biotechnology, School of Life Sciences Weihenstephan, Technical University of Munich, Freising, Germany; 12 Faculty of Medicine, Kiel University, Kiel, Germany; 13 Faculty of Medicine and Life Sciences, University of Latvia, Riga, Latvia; 14 Department of Haematology and Medical Oncology, University Medical Center Goettingen, Goettingen, Germany

**Keywords:** Vapour fixation, Phase contrast, Lung imaging, Biological techniques, Diseases, Engineering, Medical research

## Abstract

Accurate diagnosis and characterization of lung disease increasingly rely on advanced imaging modalities capable of resolving fine microstructural details while minimizing radiation exposure. Phase-sensitive computed tomography (CT), particularly propagation-based imaging (PBI), offers superior soft tissue contrast but has historically been limited by the lack of compatible fixation techniques that preserve lung architecture post-excision. We present an adapted formaldehyde (FA) vapour fixation protocol designed to maintain human-sized lungs in a physiologically inflated and morphologically stable state. This approach prevents collapse of the delicate air–tissue interfaces, a major barrier to high-fidelity phase-contrast imaging and histological correlation. Our method enables high-resolution, multiscale imaging from whole-organ PBI at 67 µm voxel size to localized subcellular synchrotron PBI at 650 nm voxel size on the same specimen, with preserved spatial relationships critical for accurate validation of imaging findings. In porcine models, FA vapour fixation maintained alveolar integrity and radiological contrast without compromising histological detail, while also avoiding the artifacts associated with liquid fixation. Crucially, the protocol allows regulation of inflation and fixation dynamics, addressing longstanding challenges in *ex vivo* lung imaging and enabling consistent specimen preparation across studies. This fixation technique supports biosafe stabilization of freshly explanted human lungs–such as those from transplant procedures creating new opportunities for translational research on pathological tissue. By bridging high-resolution radiology and histopathology, our scalable fixation protocol establishes a standardized foundation for multimodal lung imaging and offers a critical tool for advancing both fundamental lung research and clinical diagnostics.

## Introduction

Conventional X-ray imaging relies on tissue-specific differences in X-ray attenuation, which is primarily governed by absorption. Since soft tissues exhibit only weak absorption, their contrast in standard radiography is inherently poor. Moreover, X-ray absorption results in energy deposition within the tissue, which is associated with well-known side effects and limits the extent to which conventional X-ray imaging can be fully exploited, particularly in patients.

To overcome these limitations, several novel X-ray imaging techniques such as dark-field imaging and phase-contrast imaging have been developed. Unlike attenuation-based methods, these approaches exploit the wave-optical properties of X-rays. Among them, free-propagation phase-contrast imaging (PBI) offers the least complex experimental setup, as it requires only a sufficient propagation distance between the object and detector without the need for additional optical components. However, this technique demands a high degree of spatial coherence in the incident X-ray beam, restricting its application to micro-CT systems equipped with nano-focus sources or synchrotron facilities.

In phase-contrast imaging, X-rays undergo phase shifts as they traverse the sample, producing diffraction patterns that can be analyzed. These patterns have been shown to provide markedly higher sensitivity to soft tissues compared to conventional attenuation contrast. Depending on whether the recorded images correspond to near-field or far-field diffraction regimes, different algorithms are required to retrieve phase information.

In this study, we employed near-field propagation-based phase-contrast imaging (NF-PBI) with relatively large detector pixel sizes as a potential future modality for clinical lung imaging. Additionally, we investigated formalin-fixed and paraffin-embedded lung tissue specimens at higher resolution using near-field PBI (HR-NF-PBI), and at ultra high resolution using far-field PBI (UHR-FF-PBI).

Propagation-based X-ray phase-contrast imaging (PBI) is an emerging modality that enables high-resolution^1^, low-dose imaging of soft tissues^2^, offering distinct advantages for lung diagnostics^3^. Unlike conventional absorption-based imaging, PBI leverages X-ray phase shifts to detect subtle differences in tissue composition and enhance soft tissue contrast^4^.

NF-PBI provides higher resolution and soft-tissue contrast at the same X-ray dose levels then clinical CT^2^ especially in lung tissue^3^. Therefore, NF-PBI has a high potential to be used for patient thoracic CT. However, its application to human-sized lungs is currently limited to synchrotron facilities due to the requirement for coherent X-ray sources, and strongly extended propagation distances to be used in combination with clinical relevant X-ray doses. Despite these limitations, national light sources in Europe and Australia are actively pursuing the clinical translation of NF-PBI^5–7^. A critical step toward clinical adoption is the validation under conditions that reflect routine diagnostics, including reliable correlation with histopathology.

We investigated NF-PBI as a virtual lung biopsy method, to provide a non-invasive alternative to surgical tissue sampling, in cases of inconclusive clinical CT findings or contraindications to invasive procedures. Initial studies using fresh porcine lungs placed in an anthropomorphic chest phantom demonstrated that human-sized lungs could be imaged using synchrotron NF-PBI with high structural fidelity at low X-ray doses, closely approximating clinical chest CT^7–9^. However, these models lacked pathological alterations and histological correlation. In previous studies, we modeled artificial pathologies by injecting iodine-enhanced agarose gels to mimic characteristic radiological phenotypes^7^. While this approach successfully reproduced imaging features such as ground-glass opacities and tree-in-bud patterns, it did not allow for histological validation of the findings at the tissue level^7^.

To overcome these limitations, we previously examined a porcine lung affected by pneumonia. NF-PBI of the fresh lung in the chest phantom revealed infection-induced tissue alterations, but post-excision lung collapse compromised the spatial registration between imaging and subsequent histology. Formalin-fixed paraffin-embedded (FFPE) samples of this specimen exhibited extensive tissue deformation, further complicating direct correlation with imaging data (Supplementary Fig. 1). Moreover, conventional lung fixation by instillation of liquid formalin, significantly reduced the efficacy of sequential phase-contrast imaging. This highlighted the need for innovative fixation strategies that are compatible with phase-contrast imaging, preserving native aerated lung morphology.

Here we present a formaldehyde (FA) vapour fixation protocol, adapted from a historical method, that stabilizes lungs in a physiologically inflated state, while preserving air–tissue interfaces, critical for NF-PBI. FA vapour fixation enables two complementary workflows: (i) post-imaging fixation of porcine lungs for multimodal, multiscale correlation with histopathology and (ii) pre-imaging fixation of human explants under biosafety constraints. The preserved structural integrity facilitates high-fidelity, multiscale imaging, bridging PBI with microscopic histopathology. FA vapour fixation thus represents a key technical advance toward the clinical translation of NF-PBI, enabling histologically validated, high-resolution imaging of human-sized lungs at clinical relevant X-ray doses.

## Results

### Workflow and FA vapour fixation setup

**Fig. 1:**
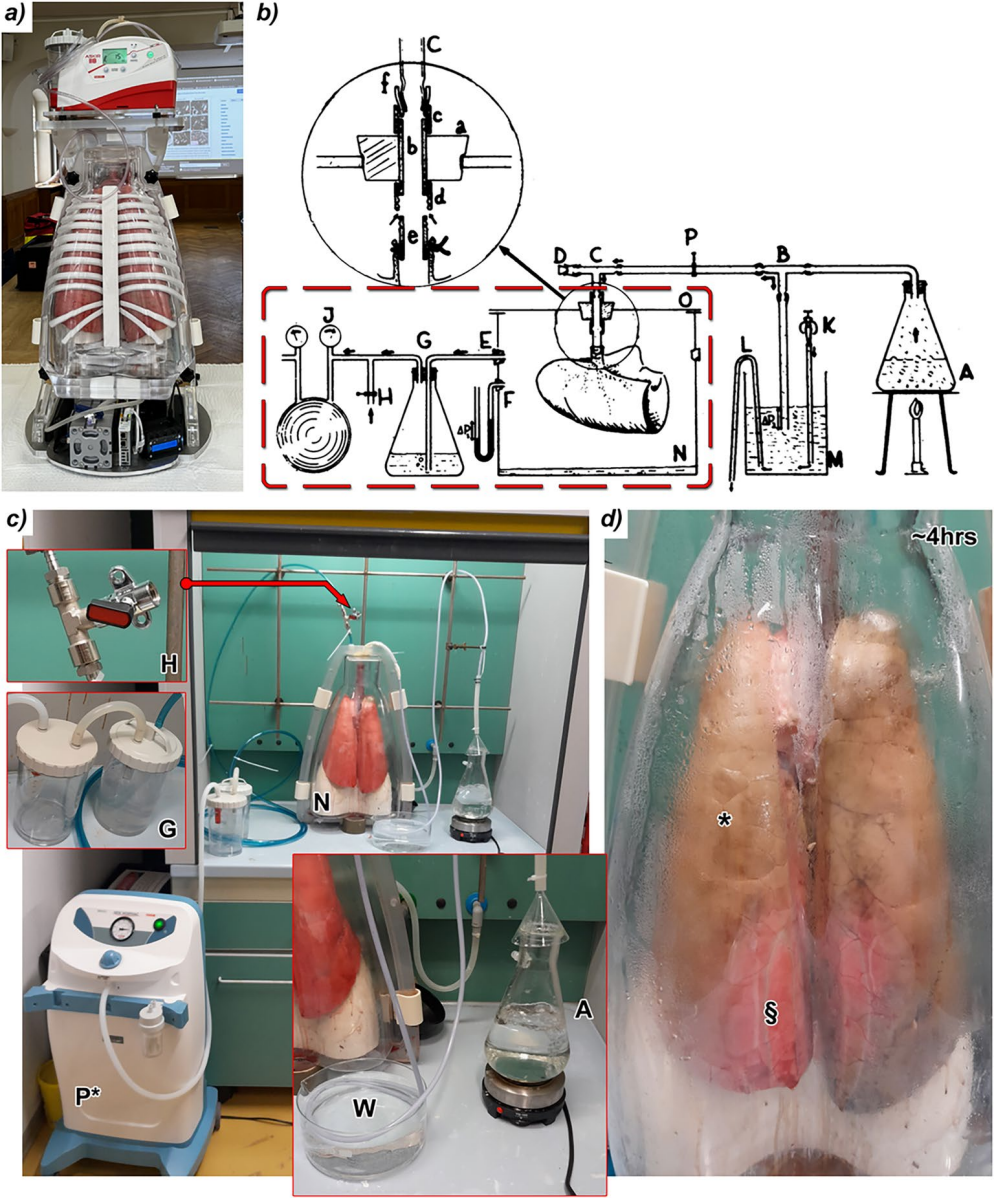
Concept and setup for formaldehyde (FA) vapour fixation of human-sized lungs **(a)** Photograph of the anthropomorphic chest phantom (ANTHONY) containing a fresh porcine lung within its inner shell, maintained in an inflated state by negative pressure generated via a pump mounted on the top of the phantom. The outer shell includes artificial bone structures mimicking a chest. **(b)** Schematic illustration of the FA vapour fixation method originally described by Weibel and Vidone^10^. Concentrated formalin (A) is heated to produce FA vapour, which is drawn into the lung’s bronchial tree through a pipe system (C) while the lung is enclosed within a sealed chamber (N) under negative pressure generated by pump (J). The red dashed rectangle outlines the functional components of the lung phantom utilized for the adapted FA vapour fixation method. **(c)** The custom vapour fixation setup used in this study placed within a chemical safety workbench. The lung (N) is housed within the inner shell of the phantom and connected to a pipe at the trachea. Negative pressure is applied via a flow pump (P*), with a valve (H) allowing for pressure adjustment. A water-filled scavenger (G) prevents FA vapour from reaching the pump. The vapour is generated by boiling formalin (A) and cooled in a water bath (W) prior to entering the lung. **(d)** Photograph of the lung following 4 hours of FA vapour fixation. The majority of the lung surface (*) exhibits a characteristic brownish colouration, indicating full penetration of the fixative. Some peripheral regions (§), in direct contact with the phantom wall, remain partially unfixed at this stage but were fully fixed by the end of the 6 h protocol.

NF-PBI of the lung leverages the strong intrinsic contrast between the soft tissue and surrounding air to achieve high-resolution images at low X-ray doses. However, the well-established lung fixation technique, based on instilling the liquid fixative formalin into the airways severely degrades image quality by replacing the air-tissue with liquid-tissue interfaces. To overcome this limitation we developed a FA vapour-based fixation method, that preserves lung architecture, while maintaining imaging contrast. The fixation setup utilizes the anthropomorphic chest phantom (ANTHONY) shown in Fig. 1a. ANTHONY comprises of (i) an inner shell that houses the lung specimen with tracheal access, (ii) an outer shell mimicking the X-ray attenuation of the chest wall and bones, (iii) a vacuum pump to create negative pressure for passive lung inflation, and (iv) a motion system to simulate breathing.

The FA vapour fixation approach was adapted from the method introduced by Weibel and Vidone^10^, as illustrated in Fig. 1b. The red-dashed components (Fig. 1b) represent the lung phantom’s features, which were repurposed for the FA vapour fixation. The complete system (Fig. 1c) was operated inside a ventilated safety workbench. A fresh porcine lung was mounted upright inside the inner shell, allowing excess fluid to collect at the bottom by gravitation. The trachea was connected to a FA vapour generator (**A**), consisting of a heating plate on which 10% formalin (4% FA) was boiled. The FA vapour was drawn into the lung via negative pressure maintained by a flow pump (P*). The FA vapour was cooled via a water bath (**W**), a three-way valve (**H**) regulated the suction, while a two-bottle scavenging unit (**G**) prevented FA vapour from entering the vacuum pump. After about 4 hours of exposure to FA vapour, the lung (Fig. 1d) exhibited a brownish hue (*), indicating FA penetration. However, tissue regions contacting the phantom wall (**§**) showed incomplete fixation due to restricted airflow, evidenced by residual redness. Full fixation was achieved after 6 hours of FA vapour exposure, followed by drainage of residual fluid from the bottom reservoir of the phantom’s inner shell.

### FA vapour fixation preserves NF-PBI image quality

**Fig. 2:**
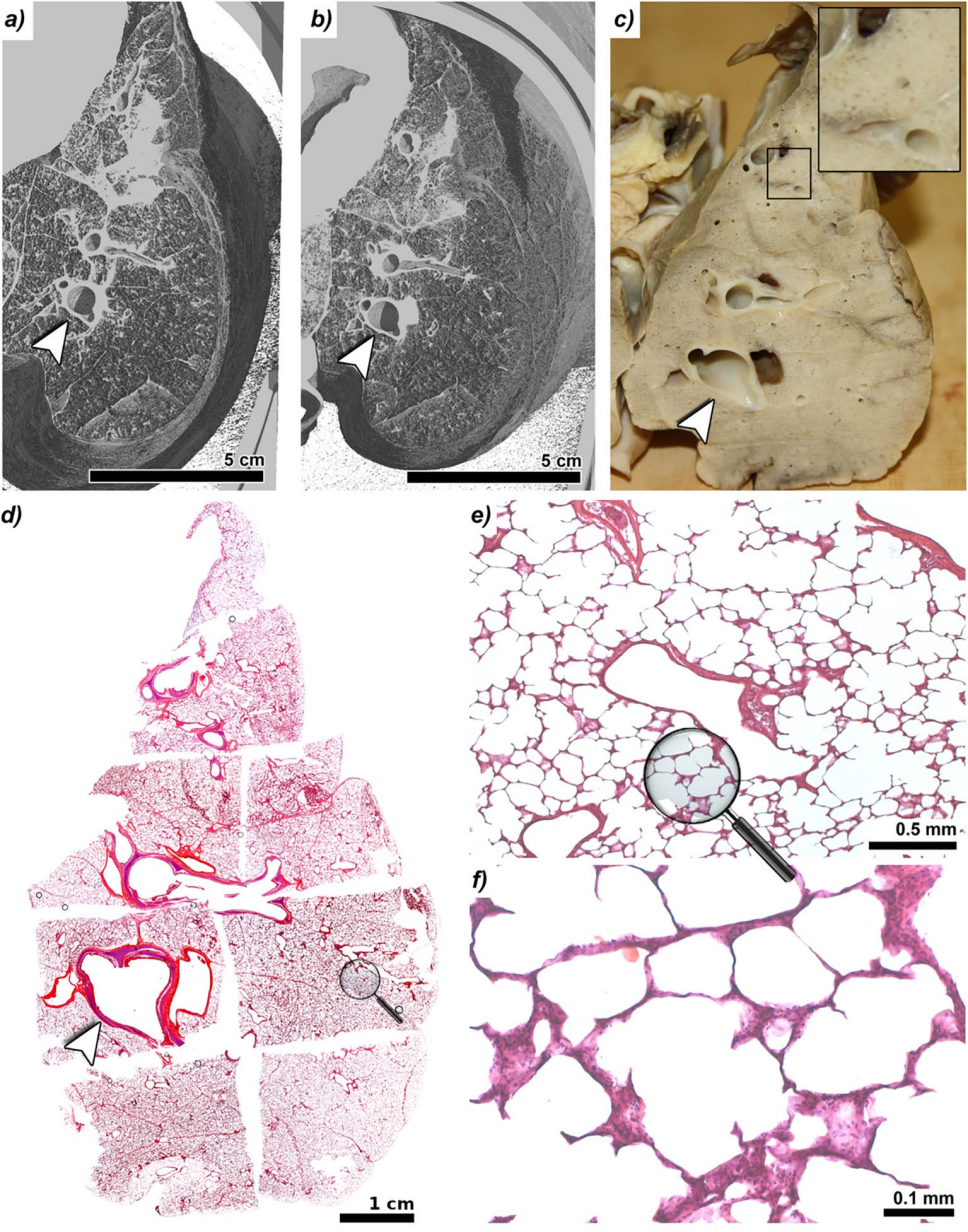
Comparative imaging of fresh and formaldehyde (FA) vapour-fixed pig lung tissue. **(a)** Three-dimensional rendering of a passively inflated fresh pig lung positioned within the anthropomorphic chest phantom, acquired using near-field propagation-based phase-contrast imaging (NF-PBI). **(b)** The same lung imaged 11.5 h later following 6 h of FA vapour fixation, without negative pressure during acquisition. No significant loss of NF-PBI image quality was observed post-fixation. **(c)** Photograph of the same lung prepared for histological analysis approximately 4 weeks after imaging. The magnified region (black rectangle) reveals successful preservation of the porous pulmonary architecture and the absence of residual liquid in the bronchial tree. The heart was removed prior to fixation to ensure consistent tissue penetration. Nine fixed paraffin-embedded (FFPE) tissue blocks were generated from the imaged slice. **(d)** Montage of the hematoxylin and eosin (H&E)-stained superficial sections of the FFPE blocks spanning the full cross-section of the fixed lung. White arrowheads in a–d indicate the same bronchus used as an anatomical reference point across modalities. Three-dimensional renderings (a and b) were virtually re-sliced to match the histological cutting plane (c) as closely as possible. **(e and f)** Higher-magnification views of a representative region (indicated by magnifying glass icons in d and e), demonstrating structural integrity and preservation of alveolar microanatomy.

A primary objective was to assess whether FA vapour fixation preserves lung structure for high-quality NF-PBI. We found that FA vapour-fixed lungs retained morphological features comparable to those of fresh specimens, with no significant loss in image quality. Figure 2a shows a three-dimensional (3D) rendering of a fresh mini-pig lung imaged in the lung phantom under passive inflation using negative pressure. The data was virtually sliced to present the image quality. The same lung was rescanned 11.5 hours later in the lung phantom without applied negative pressure, following 6 hours of FA vapour fixation and setup time (Fig. 2b). Figures 2a and 2b demonstrate, that while minor deformation was observed, overall image quality, including resolution of fine airway structures, was comparable and tissue morphology appeared largely well-preserved.

After approximately four weeks of storage in a sealed bucket at room temperature, the FA vapour-fixed mini-pig lung was sliced and processed for histology. Figure 2c shows a histological section matched to the NF-PBI datasets (Fig. 2a and 2b), confirming agreement between virtual and macroscopic slices. An approximately 10 mm thick lung tissue lamella was further dissected into smaller pieces (about 2 x 3 cm^2^), chemically dehydrated, paraffin-embedded, and hematoxylin and eosin (H&E)-stained. Figure 2d displays all H&E-stained sections from the FFPE tissue blocks of the lung tissue lamella, focusing on areas near the tissue surface. Aside from unavoidable slicing deformations, the tissue structures aligned well with those seen in NF-PBI. Magnified regions (Fig. 2e and 2f) demonstrate excellent preservation of alveolar architecture, validating the suitability of FA vapour fixation for both imaging and histopathological workflows.

### FA vapour fixation enables multiscale phase-contrast imaging

**Fig. 3:**
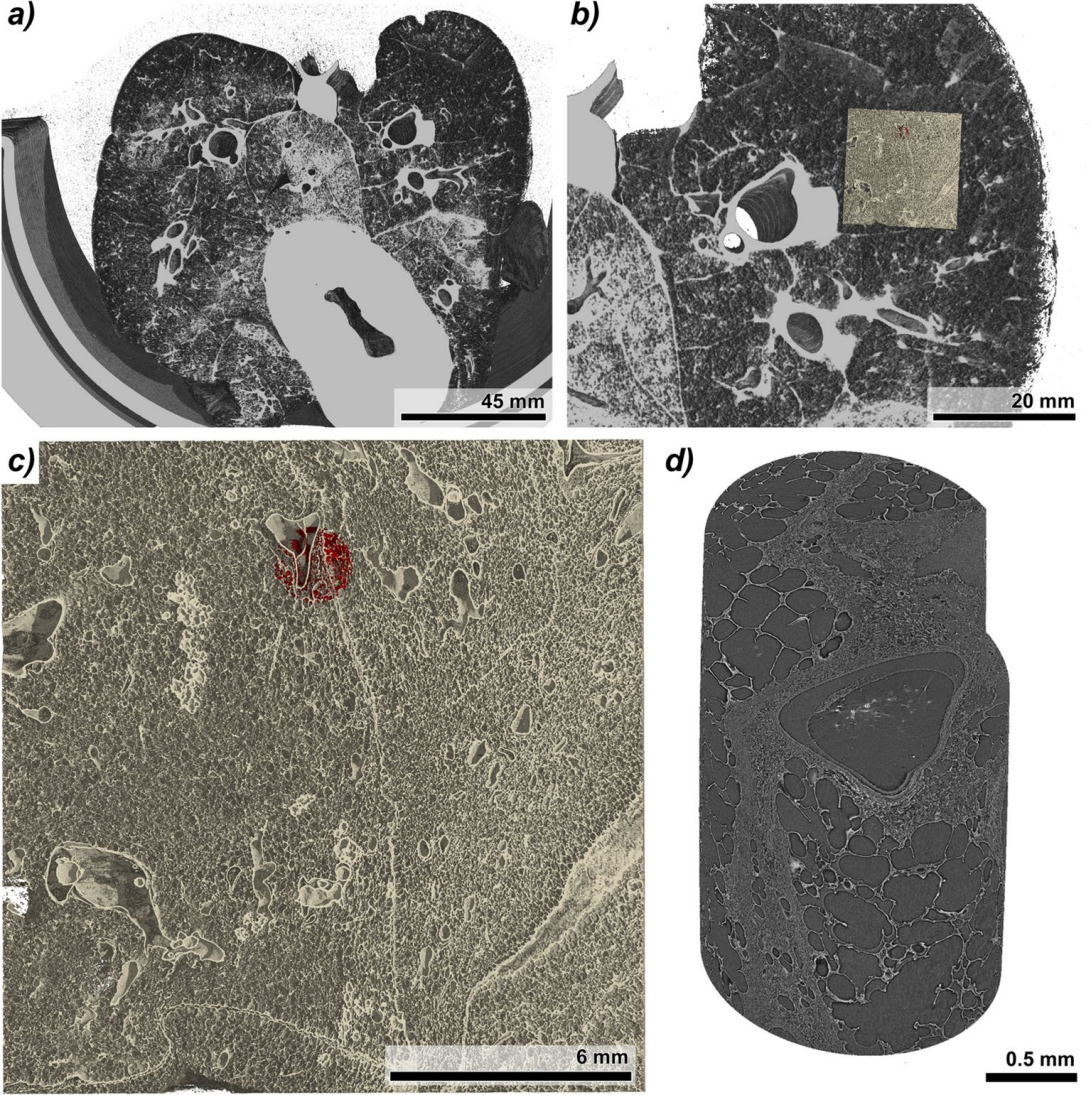
Formaldehyde (FA) vapour fixation allows multiscale hierarchical imaging of lung tissue. **(a)** Three-dimensional (3D) rendering of a FA vapour-fixed porcine lung scanned within the anthropomorphic chest phantom using near-field propagation-based phase-contrast imaging (NF-PBI) with an isotropic voxel size of 67 µm. **(b)** Magnified view showing the anatomical location of a fixed paraffin-embedded (FFPE) tissue block (highlighted in yellow) of the same lung after histological processing. **(c)** 3D mosaic rendering of the entire FFPE block (yellow colour) acquired using high resolution near-field propagation-based phase-contrast imaging (HR-NF-PBI) with an isotropic voxel size of 4.5 µm. The red volume indicates the region from which a 2 mm diameter punch biopsy was extracted. **(d)** 3D rendering of the cylindrical punch biopsy tissue sample indicated in c, imaged using ultra high resolution far-field propagation-based phase-contrast imaging (UHR-FF-PBI), with an isotropic voxel size of 650 nm, revealing fine subcellular and alveolar nanoarchitecture.

FA vapour fixation preserved the lung’s physiological inflation state, enabling hierarchical imaging and cross-scale data correlation. A 3D rendering of a NF-PBI scan of a FA vapour-fixed mini-pig lung within the lung phantom is shown in Fig. 3a. Following NF-PBI, the lung was sliced and processed for histology. FFPE tissue blocks were scanned using HR-NF-PBI at 4 µm resolution. The spatial location of one representative FFPE block, rendered in beige, was registered to the original NF-PBI dataset (rendered in grey), as shown in Fig. 3b. The rendering of the HR-NF-PBI scan of the FFPE block (Fig. 3c) reveals key anatomical features including interlobular septa, alveoli, bronchi, and vessels.

A UHR-FF-PBI scan at 0.65 µm resolution was subsequently performed on a 2 mm diameter punch biopsy from the same FFPE block, allowing visualization at cellular resolution. The biopsy location is indicated in red within the 3D HR-NF-PBI data set (beige). Figure 3d displays the 3D rendering of the UHR-FF-PBI dataset. This multiscale imaging pipeline demonstrates that FA vapour fixation is compatible with high-resolution imaging across three spatial orders of magnitude, from whole-organ (67 µm), to tissue (4 µm), and down to cellular resolution (0.65 µm).

### FA vapour-fixed lungs show well-preserved alveolar architecture in histology

**Fig. 4:**
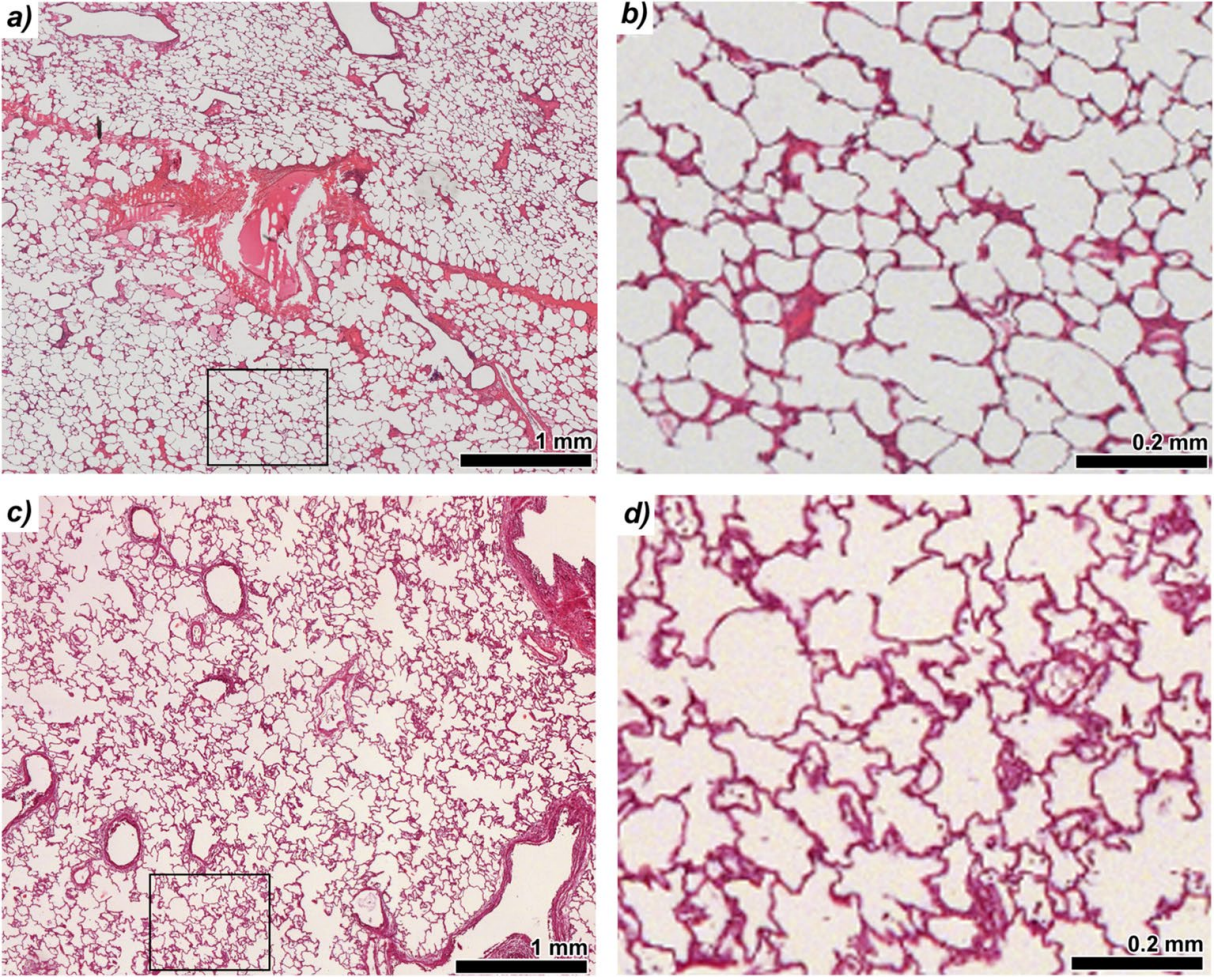
Comparison of histological quality between formaldehyde (FA) vapour fixation and conventional formalin instillation and immersion fixation of lung tissue. **(a)** Hematoxylin and eosin (H&E)-stained section from a paraffin-embedded (FFPE) tissue block of a porcine lung fixed via FA vapour. **(b)** Magnified view of the same H&E-stained section (black rectangle in a) from the FA vapour-fixed lung. The interalveolar septa appear straight, due to the air-filled state during fixation.**(c)** H&E-stained section of a porcine lung fixed by intratracheal instillation followed by full immersion in formalin. **(d)** Magnified region from c, indicated by the black rectangle, shows wavy interalveolar septa, reflecting the absence of air–surface tension during liquid instillation of fixative. Aside from this, histological quality was comparable between the two fixation methods, with no major artifacts or distortions observed.

In addition to phase-contrast-based imaging compatibility, effective preservation of tissue morphology for histology was critical. Figure 4a shows an H&E-stained slice from a FA vapour-fixed mini-pig lung, presenting the well-preserved alveolar structure. The higher magnification of the light microscopic analysis (black rectangle) demonstrates a regular acinar structure characterized by open alveolar airspaces (Fig. 4b). The interalveolar septa appear slim and exhibit a straight course. This linear morphology of the interalveolar septa indicate, that the lung was air-filled at the time of fixation, suggesting that surface tension contributed to the preserved alveolar microarchitecture. For comparison, an H&E-stained slice of a porcine lung, fixed via traditional immersion and airway filling of liquid formalin, is shown in Fig. 4c, with a higher magnification (black rectangle) shown in Fig. 4d. Alveolar airspaces appear partially collapsed, and the interalveolar septa seem thicker, exhibiting a more wavy than straight configuration, compared to the FA vapour fixed specimen. These morphological changes indicate, that the immersion-fixed lung lacked surface tension–mediated stabilization during tissue preservation.

Quantitative histological scoring of four parameters (alveolar size, homogeneity of alveolar sizes, alveolar wall thinness, and absence of ruptures) by four blinded reviewers revealed no statistically significant differences in the histological appearance of lung tissue fixed using FA vapour compared to traditional formalin instillation (Supplementary Fig. 2).

### Current set-up limitations and future optimization strategies

Effective FA vapour fixation required adequate negative pressure and dynamic regulation of suction rate to maintain appropriate passive lung inflation. As tissue stiffness increased during the fixation process, dynamic adjustment and careful monitoring of suction became essential, to prevent potential fixation artifacts, such as underinflation or tissue ruptures.

**Fig. 5:**
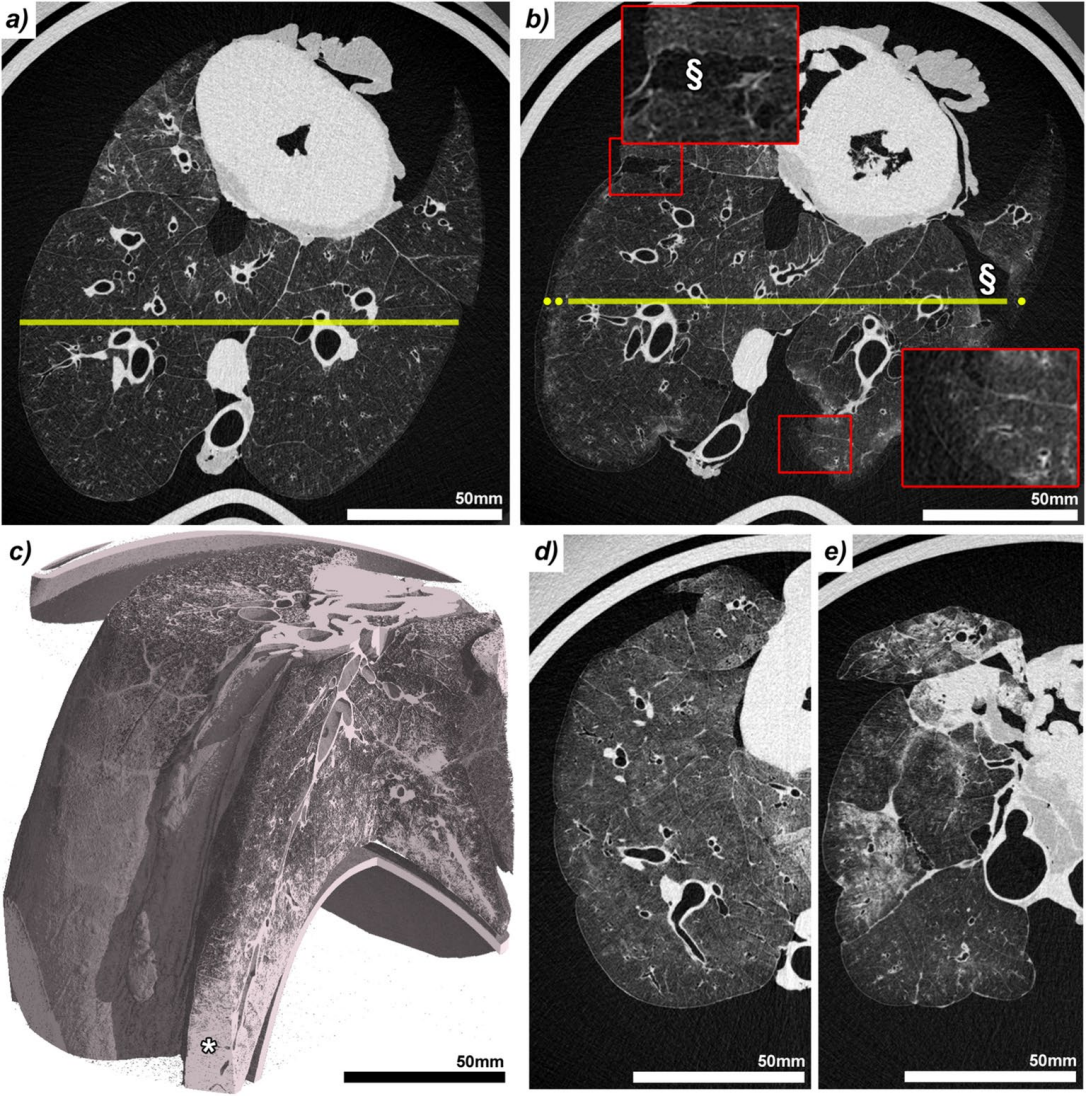
Challenges encountered during formaldehyde (FA) vapour fixation. **(a and b)** near-field propagation-based phase-contrast imaging (NF-PBI) of the same porcine lung within the anthropomorphic chest phantom before (a) and after (b) FA vapour fixation, presented as axial slices at approximately the same anatomical level. The post-fixation scan (b) shows overall volume expansion (yellow line) and surface ruptures (§), indicative of excessive strong negative pressure applied during FA vapour fixation. Additionally, the small lung size of the specific specimen made secure tracheal coupling to the FA vapour delivering tubing difficult, leading to a leaky connection and insufficient vapour delivery. The peripheral tissue remained unfixed, as shown in the inset (red rectangle, lower right corner). **(c)** Three-dimensional rendering of a post FA vapour fixation NF-PBI scan of another porcine lung in which not only FA vapour but also condensate entered the airway system during fixation. The region filled with liquid (asterisk) exhibits poor contrast in NF-PBI, underscoring the necessity of vapour-only fixation. **(d)** Axial slice of a NF-PBI scan of a lung specimen fixed under insufficient negative pressure, resulting in a compact morphology and overall surface deformation. **(e)** Axial slice of a NF-PBI scan of a lung specimen showing structural damage and regions of increased density due to condensate deposition, which may mimic pathological features in imaging.

Figure 5a shows a NF-PBI scan of a fresh mini-pig lung imaged under ideal conditions, while Fig. 5b shows the same lung post FA vapour fixation, where excessive negative pressure during fixation caused tissue rupture (§) and overexpansion (yellow line). A compromised tracheal seal of this specimen also allowed surrounding air ingress, leading to peripheral under-fixation, evident as intensity gradients in Fig. 5b (red rectangle in lower right corner). Cooling of the FA vapour occasionally caused condensation and fluid accumulation within the airways, which severely compromised soft-tissue contrast in NF-PBI (Fig. 5c), highlighting the importance of using a fixation approach, that avoids liquid deposition in the airspaces. Conversely, insufficient negative pressure during FA vapour fixation led to dense and irregular shaped lungs in the NF-PBI scans (Fig. 5d). In extreme cases, poor inflation, fluid accumulation, and tissue ruptures impaired anatomical fidelity (Fig. 5e). Additionally, as 10% formalin (4% FA) evaporated during boiling, the rising concentration of FA in the remaining solution promoted its polymerization, leading to the precipitation of paraformaldehyde.

To address these challenges in the future, we are developing an improved setup, that employs (i) a fog generator to deliver cold FA vapour, allowing for better control over FA vapour concentration and (ii) a pressure sensing module within the phantom system, to enable precise, real-time measurement of pressure during fixation.

## Discussion

We adapted the FA vapour fixation technique, originally described by Weibel and Vidone^10^, for the use with human-sized lungs in an anthropomorphic chest phantom (ANTHONY). This approach reliably preserves the lungs in an expanded state, enabling direct, high-fidelity comparisons of NF-PBI scans before and after fixation. No discernible degradation in image quality is observed, and the fixed specimens remain rigid and morphologically stable, facilitating both specimen handling and subsequent tissue collection.

The primary advantage of our FA vapor fixation method over the original approach is that the lung remains within a phantom during the fixation process. This phantom features an artificial, adjustable diaphragm^9^ and is roughly shaped like a chest cavity, ensuring realistic and controllable boundary conditions. Furthermore, due to the transportable design, the same phantom can be used for clinical and synchrotron CT imaging, enabling precise comparison of the lung’s shape and structure before and after the fixation process.

Crucially, this fixation strategy enables multiscale phase-contrast imaging and histological validation of radiological findings by preserving lung architecture *ex vivo*, a central challenge in correlating imaging data with histology. Human lungs, consisting of approximately 0.5 L of tissue embedded within several litres of air^11^, collapse rapidly upon excision. This collapse severely compromises post-excision histological assessment and hinders accurate co-registration with pre-excision imaging modalities such as clinical CT or NF-PBI.

The first generation of photon-counting detector (PCD)–based clinical CT systems is now entering the market. By directly converting incident X-rays into an electrical signal, these detectors achieve higher spatial resolution than conventional clinical CT at equal or even lower dose levels^29^. In our study, whole-lung NF-PBI was performed at a voxel size of 67 µm, surpassing the in-plane resolution of the latest ultra-high-resolution CT systems, which is typically around 200 µm^12^. While clinical photon-counting CT will certainly greatly improve the diagnosis of pathological changes within the lung, we believe that in some case an even higher resolution, which could be provided by NF-PBI, might be needed, given the fact that the alveolar size in humans is in the range of 200 to 300 µm.

A direct comparison between the X-ray doses in our study and the CTDI_vol_ index reported for CT is challenging. CTDI_vol_ is measured using standard phantoms that could not be applied here, as the maximum photon energy available at the SYRMEP beamline (40 keV) did not permit the use of the same reference setup. Nevertheless, although the detector employed for NF-PBI is based on similar technology like the once used in clinical photon-counting CT’s, NF-PBI has the potential to be more dose-efficient. This advantage arises from the enhanced soft-tissue contrast provided by propagation-based phase contrast, the use of a monochromatic X-ray beam, and the reduced decay of phase contrast with increasing photon energy compared to attenuation-based CT. Importantly, this implies that patient imaging could be performed at higher photon energies, such as those used in clinical CT systems, thereby further reducing radiation dose^30^.

However, it remains imperative that imaging findings are rigorously validated. At this high resolutions, that can virtually only be done using subsequent histological or complementary analytical techniques. To enable such a strategy was the main goal of the here presented study.

Knudsen et al.^13^ emphasized the importance of controlled lung inflation during fixation to enable meaningful quantitative comparison across imaging modalities. Our modified FA vapour-fixation protocol addresses this by enabling the regulation of flow rates throughout the fixation process. The historical application of FA vapour for fixation^10^ was also built upon the work by King et al.^14^ for high-resolution CT, who demonstrated the detrimental imaging effects of inadvertent liquid infiltration.

Further we observed minor shrinkage of the lung tissue post FA vapour fixation, consistent with previous reports by Weibel and Vidone^10^, who reported linear shrinkage of up to 18%. Especially, in cases of pathological changes like lung fibrosis or emphysema, the deformation of the specimen will be non-linear. Further quantitative studies are needed to characterize this effect across specimens and to evaluate whether slight overinflation during fixation could mitigate shrinkage without inducing structural damage.

Our histological scoring results indicated no significant differences between our new FA vapour fixation approach compared to the filling of the airways with FA and immersing the entire lung in FA, which we consider to be the established approach. This indicates that our new protocol is at least as good as the conventional method. We even see the tendency of higher scores for the FA vapour approach and a more physiological appearance of the alveolar wall, most likely due to the better controlled pressure during the fixation process and the differences in surface tension.

In pilot experiments, agarose gel mixed with iodine was injected into healthy porcine lungs within the lung phantom to simulate pathological features in NF-PBI scans^7^. While this approach successfully mimicked radiologic patterns of lung disease, it lacked histological validation. Moreover, anatomical differences, particularly in bronchial branching and cartilage content, limit the extent to which porcine models recapitulate human lung pathology. Access to freshly explanted, pathologically altered human lungs remains a limiting factor. However, clinical scenarios such as lung transplantation could provide valuable opportunities. Our portable fixation setup is well suited for rapid stabilization of such specimens, enabling subsequent high-resolution imaging and histopathological analysis.

Beyond technical validation, FA vapour fixation offers new opportunities for investigating lung disease across spatial scales and modalities. Its compatibility with synchrotron-based and micro-CT imaging allows detailed structural mapping of disease models, such as fibrosis, pneumonia, or emphysema, while preserving spatial context for histopathological interpretation. This is particularly valuable in settings like transplantation, where human explanted lungs can be fixed under biosafety conditions immediately after resection, supporting retrospective or prospective correlation of imaging features with histological diagnoses.

Human lungs have been studied in toto *ex vivo* with hierarchical phase contrast CT as for instance reported by Xian et al.^25^. However, for those studies the lung was filled with formalin and later chemically dried with ethanol and scanned immersed in ethanol. These experiments aim to provide high quality structural information of whole human organs and use long scanning times and high X-ray doses. In contrast we developed a fixation approach which allows to scan the lung at clinical relevant X-ray dose levels and provide the ability to correlate the imaging findings with subsequently performed histology.

In this study, we demonstrate for the first time that NF-PBI can be applied to whole-lung CT in human-sized lungs, achieving clinically relevant dose levels while providing substantially higher spatial resolution. In contrast, UHR-FF-PBI has previously been successfully employed only for imaging smaller tissue specimens under various conditions, including agarose-^26^, resin-^27^, and paraffin-embedded^28^ samples. Especially in the latter case high-resolution PBI offers to scan standard formalin-fixed and paraffin-embedded tissue without additional staining, which eases correlation of the 3D imaging findings with histology as demonstrated for instance by Reichmann et al.^3^. Typically these approaches start with small tissue specimens. Our fixation protocol on the other hand allows to put the histology results into perspective of the macroscopic structure of the entire lung, thereby allowing to use the NF-PBI scans to target specific regions for subsequent histological analysis.

In summary, our adapted FA vapour fixation method provides a practical and scalable solution for preserving lung architecture during multimodal and multiscale imaging. When integrated with high-resolution PBI, it opens the possibility for comprehensive, quantitative analyses of lung microstructure across healthy and diseased states. Future work will focus on applying this method to human lungs with confirmed pathology and on refining fixation protocols to further reduce tissue distortion. As imaging techniques continue to advance, standardized *ex vivo* preparation protocols, such as the one presented here, will be essential for integrating data across scales and modalities in lung research and for facilitating clinical translation.

## Methods

### Specimens and sample preparation

Lungs from five adult domestic pigs (body mass $\approx$ 80 kg) were obtained post-mortem from a licensed slaughterhouse as by-products of food production. Additionally, lungs from two adult mini-pigs (body mass $\approx$ 40 kg) were obtained from the Animal Research Center (ARC) of the Technical University of Munich (TUM) as part of a licensed sarcoma study, after study completion (animal permit number: 55.2-2532.Vet_02-18-33). No animals were sacrificed specifically for this study.

All lungs were stored at $-20^{\circ }$C for several days to weeks and transported frozen to the SYRMEP beamline (Elettra Synchrotron, Trieste, Italy). Prior to imaging, samples were defrosted at room temperature over $\approx$ 12 h to minimize ice crystal formation. Residual connective tissue, including fat between lobes and diaphragm remains, was carefully removed. The tracheae were trimmed as needed to fit the lungs into the anthropomorphic chest phantom, based on the estimated size of the non-inflated lungs. If a lung appeared large, a greater portion of the trachea was removed, for smaller lungs, less trimming was performed. Where necessary, visually apparent surface defects of the lungs were sutured using surgical suture material and sealed with cyanoacrylate glue (Supplementary Fig. 3). The fresh lungs were examined using NF-PBI in the anthropomorphic chest phantom under passive inflation. One additional lung of an adult domestic pig was excluded from NF-PBI and was only used for classic lung fixation by intratracheal instillation and immersion in formalin.

### Anthropomorphic chest phantom (ANTHONY)

To simulate potential thoracic imaging of patients, porcine lung specimen were positioned within a custom-built anthropomorphic chest phantom (**ANTHONY**=**ANT**hropomorphic p**H**antom f**O**r lu**N**g tomograph**Y**, MiMEDA GmbH, Germany), developed based on the ArtiCHEST phantom^15^. The phantom comprises an inner shell enclosing the lung, connected via a tracheal tube to the atmosphere. The accessible trachea is a critical feature of the phantom, enabling not only FA vapour fixation but also facilitating procedures such as bronchoscopy and targeted tissue sampling. The lung was carefully positioned inside the inner shell and fastened to the tracheal connector using a cable tie. To prevent tissue drying during the experiment, a thin layer of coconut oil was applied to the lung surface before the inner shell was closed. The phantom incorporates a rigid artificial diaphragm that can be positioned at three different levels to accommodate variations in lung size. If the lung appeared small upon visual assessment, the highest diaphragm position was selected and for larger lungs, the lowest position was used. The inner phantom also features a silicone seal. Negative pressure was applied to the sealed inner shell using the cable-operated vacuum pump Hospivac H400 (CA-MI S.r.l., Italy). The device allows a maximum vacuum of –90 kPa, and a maximum flow rate of 90 L/min to induce passive lung inflation with ambient air.

The current system does not allow for precise measurement of static pressure within the phantom. While the design helps prevent over-inflation of larger lungs, suction levels had to be carefully regulated for smaller lungs to avoid over-expansion. Lung inflation was visually assessed during the application of negative pressure, which was manually adjusted to achieve adequate expansion. The transparent acrylic casing of the phantom enabled continuous visual monitoring of lung volume and inflation. The sealed inner shell was inserted into an outer shell, designed to be mounted upright in synchrotron setups or to be placed horizontally within clinical CT scanners. However, only the synchrotron setup was used in this study. Outer shell variants are available with or without artificial bone structures to approximate chest wall attenuation. In this study, the variant without artificial bone structures was used for imaging. Further the outer shell allows for the connection of a vacuum pump and is also capable of integrating a breathing simulation mechanism, although this functionality was not employed in the present study (Supplementary Fig. 3). After NF-PBI scanning, solely the inner shell of the phantom was used for fixation with FA vapour, followed by a second NF-PBI scan under identical imaging parameters to assess fixation effects.

### Near-field propagation-based phase-contrast imaging (NF-PBI)

NF-PBI of the porcine lungs in the anthropomorphic lung phantom was conducted at the SYRMEP beamline of the synchrotron Elettra (Trieste, Italy)^16^ using a monochromatic X-ray beam of 40 keV ($\Delta$E $\approx$50 eV) with a 10.7 m sample-to-detector distance. A single-photon counting detector (Varex Imaging, USA; formerly Direct Conversion AB, Sweden) with a nominal pixel size of 100 x 100 µm^2^ was used. Given the divergence of the beam this resulted in an effective pixel size of 67 µm. Aluminium filters of 3.75 mm or 7.75 mm thickness were employed, and the detector minimal energy threshold was set to 24 keV. The beam dimension at the phantom position was 144 x 4.6 mm^2^.

Each scan consisted of 3600 angular spaced projections over 360°, acquired in an off-centre rotation mode. The exposure time per projection was 10 ms, yielding a total acquisition time of 36 s per full rotation. To capture extended lung volumes, up to 94 vertically overlapping scans (1.6 mm increment) were acquired and stitched using a custom Python script. Entrance radiation exposure was estimated via thermoluminescence dosimeters as $\approx$ 43 mGy and $\approx$ 23 mGy for 3.75 mm and 7.75 mm filters, respectively.

### Formaldehyde (FA) vapour fixation

Lungs subjected to FA vapour fixation were prepared to preserve lung architecture while maintaining the physiological air-tissue interface essential for PBI. This method was adapted from the classical approach introduced by Weibel and Vidone^10^ and integrated with the anthropomorphic chest phantom ANTHONY used for NF-PBI.

The inner shell of the phantom, containing the fresh porcine lung, was used for fixation. Following imaging, lungs were maintained in an inflated state within the inner shell of the anthropomorphic phantom by applying negative pressure using the cable-operated vacuum pump Hospivac H400 (CA-MI S.r.l., Italy). The device allows a maximum vacuum of 90 kPa, and a maximum flow rate of 90 L/min. Lung inflation was assessed visually during the application of negative pressure to ensure adequate expansion, facilitated by the transparent inner shell, which enabled continuous monitoring. The vacuum level was carefully manually adjusted during the FA vapour fixation to ensure stable lung inflation while preserving tissue integrity. The lung specimen was positioned upright, allowing gravitational drainage of residual fluids. The trachea was connected to a FA vapour generator comprising a heating plate boiling 10% formalin (4% FA). This vapour was cooled via a water bath to regulate temperature and prevent condensation prior to entry into the lung via the trachea. Negative pressure was maintained within the inner shell by a flow pump, drawing the FA vapour passively into the lung airways to ensure uniform exposure. A three-way valve controlled vapour suction, while a two-bottle scavenging system prevented vapour ingress into the vacuum pump, ensuring safety and system integrity. The entire fixation apparatus operated within a ventilated safety workbench to mitigate FA exposure risks. Lungs were exposed to FA vapour for approximately six hours, during which tissue gradually acquired a brownish hue indicative of FA penetration. At the conclusion of the fixation protocol, only minimal lung shrinkage was observed as the suction rate of the pressure pump was gradually reduced. Fixed lungs were subsequently stored at room temperature in sealed buckets without additional fixatives for up to four weeks before histological processing.

### Classical lung fixation via formalin instillation

For comparison with the FA vapour-based fixation protocol, one additional lung from an adult domestic pig was fixed using the conventional method of intratracheal instillation and immersion of formalin. Following thawing and careful preparation of the lung, 10% formalin (containing 4% formaldehyde) was instilled into the airways. Subsequently, the lung was fully submerged in the same fixative to ensure complete tissue penetration. This classical method served as a benchmark for histological preservation and structural integrity and was directly compared to the FA vapour fixation approach described above. Tissue sections from both fixation methods were processed identically for paraffin embedding, sectioning, hematoxylin and eosin (H&E) staining, and blinded histological assessment, as described for the FA vapour fixed specimen.

### Histology and scoring of histological quality

Following the post-fixation NF-PBI acquisition, lungs were removed from the phantom and stored in sealed buckets without additional fixative. After 2–4 weeks, lungs were sectioned into 5 mm slices, with one representative slice taken per lung.

Lungs were processed into formalin-fixed, paraffin-embedded (FFPE) blocks using the institutional standard histopathology workflow. Lung sections were first cut into smaller segments of approximately $\approx$ 2 x 3 cm^2^ to ensure compatibility with embedding cassettes and to allow uniform reagent penetration during processing. Dehydration was carried out in a graded ethanol series, with sequential incubations of 3 hours each in 60%, 75%, 96%, and 100% ethanol. This was followed by a 3-hour incubation in xylene to remove residual ethanol and prepare the tissue for paraffin infiltration. Samples were then transferred to molten paraffin wax and kept in the paraffin until embedding. During paraffin embedding, the formation of air bubbles was carefully avoided, as even small inclusions can lead to significant artifacts in phase-contrast imaging. FFPE blocks were then cooled and stored at room temperature until sectioning.

The same standardized embedding protocol was applied to both traditional formalin-instilled and FA vapour-fixed specimens. For clarity throughout the manuscript, the term “FFPE” is used to refer to all paraffin-embedded samples. However, it should be noted that only the conventionally instilled samples were fixed in liquid formalin, whereas the others were preserved using FA vapour, derived from boiling 10% formalin (4% FA). The slicing and embedding process of each specimen was carefully documented photographically to facilitate the subsequent localization of histological data within the NF-PBI scans. The anatomical level and angle of each cut relative to the whole lung, along with identifiable anatomical landmarks, such as major bronchi and their spatial patterns, were used for orientation. FFPE tissue blocks were sectioned at 2 µm thickness using a rotary microtome and afterwards mounted on a stretched water bath to ensure smooth, wrinkle-free adhesion. The superficial sections encompassing the full cross-section were stained with H&E according to the institutional standard protocol, following the method described by Cardiff et al.^17^. Mosaic microscopy images were obtained at 5x magnification, to cover the entire histological specimen, and higher-resolution images at 20x magnification were all obtained using an Axiovert 200 inverted microscope (Zeiss, Germany).

To evaluate the preservation of pulmonary microarchitecture following FA vapour fixation, a quantitative histological scoring system was developed and applied. Four morphological criteria (alveolar size, homogeneity of alveolar sizes, alveolar wall thinness, and absence of ruptures) were independently assessed by four blinded observers. Representative H&E-stained sections from the FA vapour fixation group (n=4) and the formalin instillation group (n=4) were selected from matched anatomical regions of the lungs for histological scoring. Scoring was performed on these sections to enable direct comparison between the two fixation methods: traditional formalin instillation (“filled”) and FA vapour-based fixation (“vapour”). Each criterion was rated on a 4-point ordinal scale from 0 (poor) to 3 (excellent), in 0.5-point increments. Slide images were anonymized and randomized to eliminate bias during assessment. This custom scoring approach enabled structured, reproducible comparisons of histological quality across fixation methods.

### High-resolution near-field propagation-based phase-contrast imaging (HR-NF-PBI)

FFPE lung tissue blocks were scanned at the SYRMEP beamline using a polychromatic (“white”) X-ray beam at a 500 mm sample-to-detector distance. Imaging was performed using a 16-bit water-cooled sCMOS camera (Hamamatsu C11440-22C ORCA-Flash 4.0 v2), acquiring 3600 angular distributed projections with 50 ms exposure time, resulting in an isotropic voxel size of 4.5 µm. Scanning was performed in 360° offset mode, resulting in a scan time of 180 s. A 0.5 mm Si filter was used, resulting in a mean beam energy of 19.6 keV. To cover the peripheral region of interest near the block surface, mosaic acquisitions with 2 x 3 or 2 x 4 tiles were conducted.

### Ultra-high-resolution far-field propagation-based phase-contrast imaging (UHR-FF-PBI)

2 mm diameter cylindrical punch biopsies of FFPE lung tissue blocks were obtained with disposable dermal biopsy punches (Kai Industries Co. LTD., Japan) with a custom-built ejection tool for gentle extraction of the specimen. Punches were scanned using the high-throughput tomography (HiTT) setup at the EMBL P14 beamline (PETRA III, DESY, Hamburg, Germany), as described by Albers et al.^18^. An X-ray microscope with a 10x objective coupled to a PCO.edge 4.2 sCMOS camera was used, resulting in an effective pixel-size of 0.650 µm. Imaging was performed with a monochromatic X-ray beam with a photon energy of 18 keV, acquiring 3600 projection images over a rotation angle of 360° with an offset rotation axis to increase the lateral field of view (FOV).

Each sample was scanned at four sample-to-detector distances (73, 77, 83, and 92 mm) to enable phase retrieval using a contrast-transfer-function (CTF) algorithm. An exposure time of 10 ms per projection was used resulting in a total exposure time of 144 s per acquisition. For each specimen three adjacent scans with a vertical offset of 0.9 mm were acquired resulting in a FOV of approximately 2.6 x 2.6 x 3.1 mm^3^. Phase retrieval and reconstruction were performed using the in-house TOMO-CTF software with a $\delta /\beta$ ratio of 10. Reconstructions were stitched with NRStitcher.

### Software and statistics

All data sets acquired at the SYRMEP beamline were reconstructed using the SYRMEP Tomo Project software (STP Version 1.6.3)^19^. A single distance phase retrieval algorithm developed by Paganin et al.^20^ was used with a $\delta /\beta$ ratio of 2000 for whole lungs and 100 for FFPE-tissue. Preprocessing was done in Fiji (NIH)^21^. For stitching of the mosaic white-beam acquisition NRStitcher^22^ was applied. Vertical stitching of the whole lung PBI scans was achieved with a custom made Python script based on scikit-image phase cross-correlation function^23^. Statistical analyses were performed using the Python packages seaborn^24^ and statanot (https://pypi.org/project/statannot). Histological scoring data are presented as box plots showing the median, interquartile range, and minimum and maximum values. Statistical differences between groups were assessed using a two-tailed Welch’s t-test with significance defined at $P < 0.05$. Volume rendering of the three-dimensional data sets were performed using VGStudio Max v3.1 (Volumegraphics, Germany). Figures were generated with Photoshop 6.0 (Adobe, USA).

## Supplementary Information

Supplementary Information.

## Data Availability

The datasets generated and analysed in the context of this study are available from the corresponding author upon reasonable request.

## Materials availability

Materials, such as sealed containers for storing FA vapour–fixed lung specimens, are available from the corresponding authors upon reasonable request.

## Code availability

The Python code used in this study was developed for the specific setup and workflow used. Therefore, it may have limited applicability to other configurations or use cases. However, the code is available from the corresponding authors upon reasonable request.
