# Supplementary material for: Formaldehyde vapour fixation enables multiscale phase-contrast imaging and histological validation of human-sized lungs
Source: Sci Rep. 2025 Oct 20;15:36475. doi: 10.1038/s41598-025-23903-1 (PMC12537873; doi:10.1038/s41598-025-23903-1)
Supplement: Supplementary file 1 — Supplementary Information. [file 41598_2025_23903_MOESM1_ESM.doc]

Supplemental Material

Formaldehyde vapour fixation enables multiscale phase-contrast imaging and histological validation of human-sized lungs

Christian Dullin^1,2,3,4,5*^, Johanna Reiser^4,5^***^†^***, Willi L. Wagner^4,5,6^, Elena Longo^3^, Marko Prašek^3^, Adriano Contillo^3^, Nicola Sodini^3^, Diego Dreossi^3^, Paola Confalonieri^7,8^, Francesco Salton^7,8^,

Marco Confalonieri^7,8^, Elisa Baratella^9^, Maria Assunta Cova^9^, Claudia V. Benke^4,5^, Md Motiur Rahman Sagar^2^,

Lorenzo D’Amico^3,10^, Jonas Albers^10^, Angelika Svetlove^10^, Elizabeth Duke^10^, Tatiana Flisikowska^11^, Krzysztof Flisikowski^11^, Mark O. Wielpütz^4,5,6^, Ju¨rgen Biederer^4,5,12,13^,

Hans-Ulrich Kauczor^4,5^, Frauke Alves^1,2,14^, Fabrizio Zanconati^8^, Giuliana Tromba^3^

^1*^Department of Clinical and Interventional Radiology, University Medical Center Goettingen, Goettingen, Germany.

^2^Translational Molecular Imaging, Max-Planck-Institute for Multidisciplinary Sciences, Goettingen, Germany.

^3^Elettra-Sincrotrone Trieste S.C.p.A., Basovizza, Italy.

^4^Department of Diagnostic and Interventional Radiology, University Hospital Heidelberg, Heidelberg, Germany.

^5^Translational Lung Research Center Heidelberg (TLRC-H), German Center for Lung Research (DZL), Heidelberg, Germany.

^6^Diagnostic Radiology and Neuroradiology, University Medicine Greifswald, Greifswald, Germany.

^7^Department of Pulmonology, University Hospital of Cattinara, University of Trieste, Trieste, Italy.

^8^Department of Medical, Surgical and Health Sciences, University Hospital of Cattinara, University of Trieste, Trieste, Italy.

^9^Department of Radiology, University Hospital of Cattinara, University of Trieste, Trieste, Italy.

^10^European Molecular Biology Laboratory (EMBL), Hamburg Unit c/o DESY, Hamburg, Germany.

^11^Department of Molecular Life Sciences, Chair of Livestock Biotechnology, School of Life Sciences Weihenstephan, Technical University of Munich, Freising, Germany.

^12^Faculty of Medicine, Kiel University, Kiel, Germany.

^13^Faculty of Medicine and Life Sciences, University of Latvia, Riga, Latvia.

^14^Department of Haematology and Medical Oncology, University Medical Center Goettingen, Goettingen, Germany.

*Corresponding author(s). E-mail(s): [christian.dullin@protonmail.com;](mailto:christian.dullin@protonmail.com) Contributing authors: [johanna.s.reiser@gmail.com;](mailto:johanna.s.reiser@gmail.com) [willi.wagner@med.uni-greifswald.de;](mailto:willi.wagner@med.uni-greifswald.de) [elena.longo@elettra.eu;](mailto:elena.longo@elettra.eu) [marko.prasek@elettra.eu;](mailto:marko.prasek@elettra.eu) [adriano.contillo@elettra.eu;](mailto:adriano.contillo@elettra.eu) [nicola.sodini@elettra.eu;](mailto:nicola.sodini@elettra.eu) [diego.dreossi@elettra.eu;](mailto:diego.dreossi@elettra.eu) [paola.confalonieri@asugi.sanita.fvg.it;](mailto:paola.confalonieri@asugi.sanita.fvg.it) [francesco.salton@asugi.sanita.fvg.it;](mailto:francesco.salton@asugi.sanita.fvg.it) [marco.confalonieri@asugi.sanita.fvg.it;](mailto:marco.confalonieri@asugi.sanita.fvg.it) [elisa.baratella@asugi.sanita.fvg.it;](mailto:elisa.baratella@asugi.sanita.fvg.it) [m.cova@fmc.units.it;](mailto:m.cova@fmc.units.it) [claudia.benke@med.uni-heidelberg.de;](mailto:claudia.benke@med.uni-heidelberg.de) [motiur.sagar@mpinat.mpg.de;](mailto:motiur.sagar@mpinat.mpg.de) [lorenzo.damico@embl-hamburg.de;](mailto:lorenzo.damico@embl-hamburg.de) [jonas.albers@embl-hamburg.de;](mailto:jonas.albers@embl-hamburg.de) [angelika.svetlove@embl-hamburg.de;](mailto:angelika.svetlove@embl-hamburg.de) [elizabeth.duke@embl-hamburg.de;](mailto:elizabeth.duke@embl-hamburg.de) [tatiana.flisikowska@tum.de;](mailto:tatiana.flisikowska@tum.de) [krzysztof.flisikowski@tum.de;](mailto:krzysztof.flisikowski@tum.de) [mark.wielpuetz@med.uni-greifswald.de;](mailto:mark.wielpuetz@med.uni-greifswald.de)

[juergen.biederer@uni-heidelberg.de;](mailto:juergen.biederer@uni-heidelberg.de)

[Hans-Ulrich.Kauczor@med.uni-heidelberg.de;](mailto:Hans-Ulrich.Kauczor@med.uni-heidelberg.de) [falves@gwdg.de;](mailto:falves@gwdg.de) [fabrizio.zanconati@asugi.sanita.fvg.it;](mailto:fabrizio.zanconati@asugi.sanita.fvg.it) [giuliana.tromba@elettra.eu;](mailto:giuliana.tromba@elettra.eu)

***^†^***These authors contributed equally to this work.


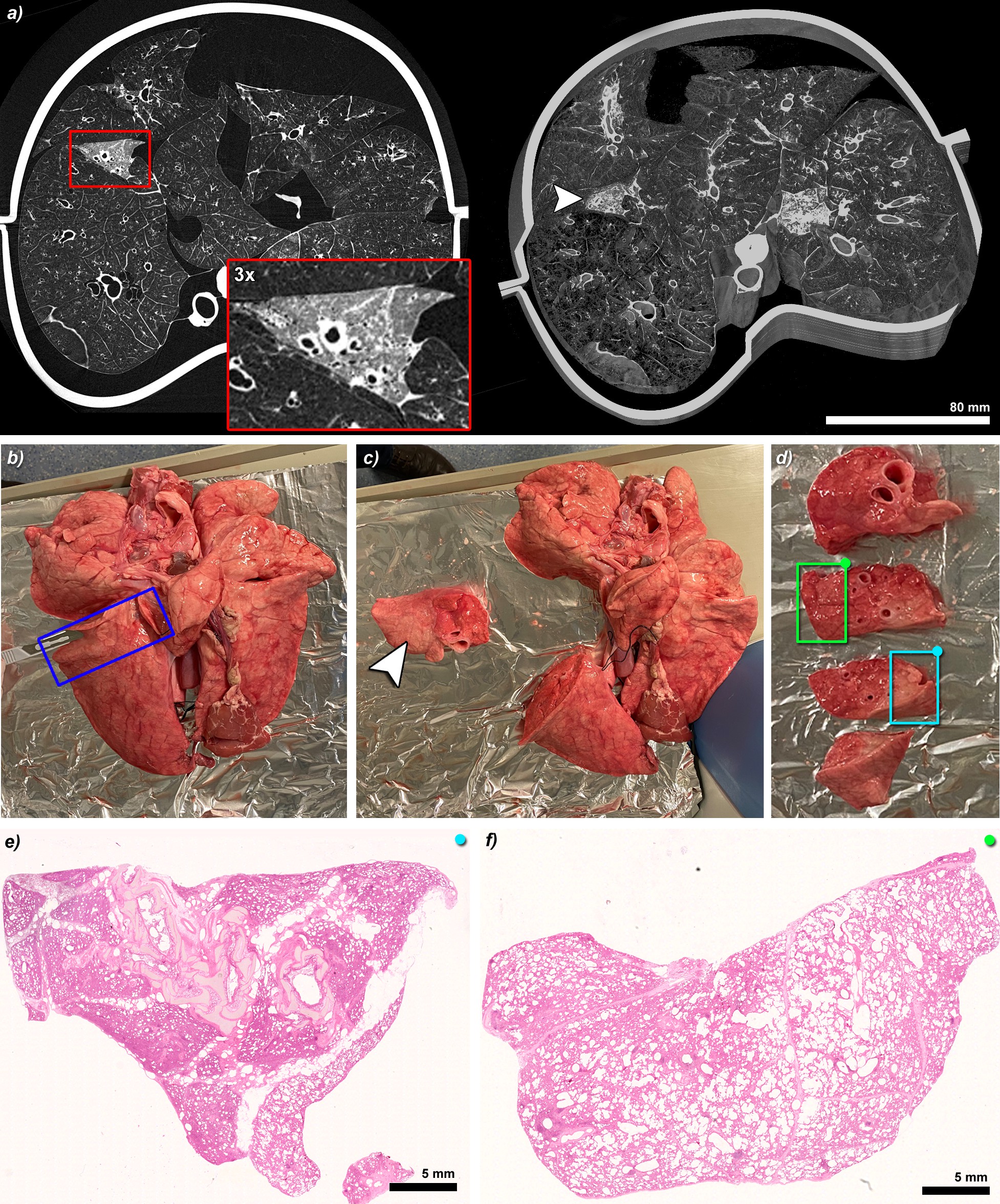


**Fig. S1 Near-field propagation-based phase-contrast imaging (NF-PBI) and histology of a lung from a pig recovered from pneumonia.**

**(a)** NF-PBI scan of a porcine lung within the anthropomorphic chest phantom, shown as a single axial slice (left) and three-dimensional rendering (right). The pig had recovered from pneumonia, and localized changes are evident in one lobe (magnified region, indicated by a red rectangle). **(b)** Photograph of the collapsed lung post-removal from the phantom. The blue rectangle marks the region selected for further analysis. **(c)** Macroscopic examination revealed a denser, paler region in the targeted area (white arrowhead). **(d)** Tissue lamellae (*≈* 1 cm thick) extracted from the region of interest. Rectangles indicate pathologically altered (turquoise) and presumed healthy regions (green), which both were processed into formalin-fixed and paraffin-embedded (FFPE) tissue blocks. **(e and f)** Hematoxylin and eosin (H&E)-stained sections from the affected (e) and control (f) FFPE tissue blocks. The pathological tissue corresponds morphologically to the dense area identified in the NF- PBI scan (red rectangle in a), though histological quality was poor overall, with signs of damage, collapse, and distortion.


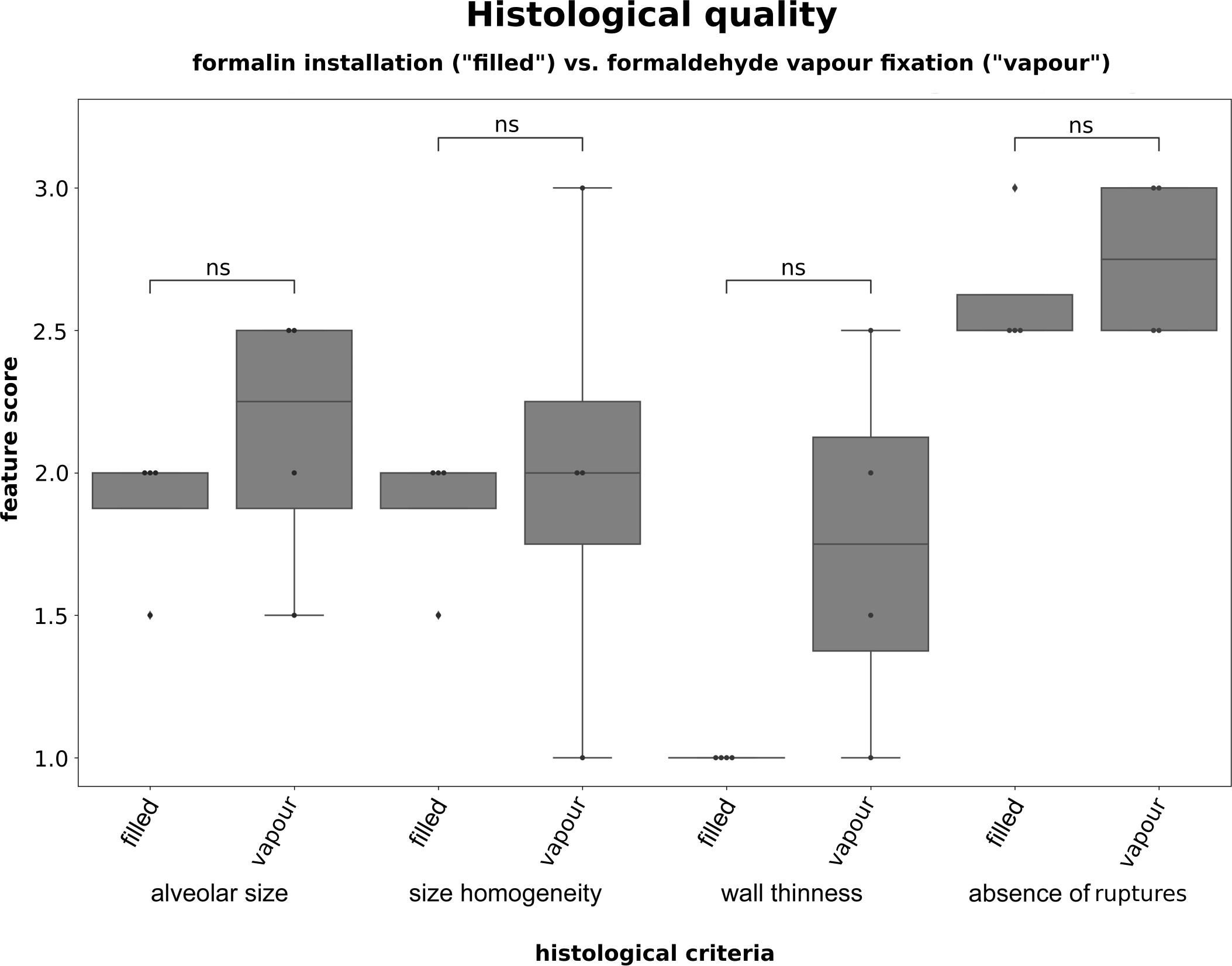


**Fig. S2 Scoring of histological quality following formaldehyde (FA) vapour and classical fixation.** Quantitative assessment of four histological criteria (alveolar size, homogeneity of alveolar sizes, alveolar wall thinness, and absence of ruptures) was performed by four independent, blinded observers using a 4-point scale (from 0 = poor to 3 = excellent). Results compare classical fixation via formalin instillation (”filled”) with the FA vapour fixation approach (”vapour”). While FA vapour fixation scored slightly higher on average, differences were not statistically significant. A two-tailed Welch’s t-test was performed, with significance defined at P < 0.05. Data are presented as box plots depicting the median, interquartile range, and minimum and maximum values. Representative hematoxylin and eosin (H&E)-stained slices were selected from the vapour fixation (n = 4) and instillation fixation (n = 4) groups for scoring. ns = not significant.


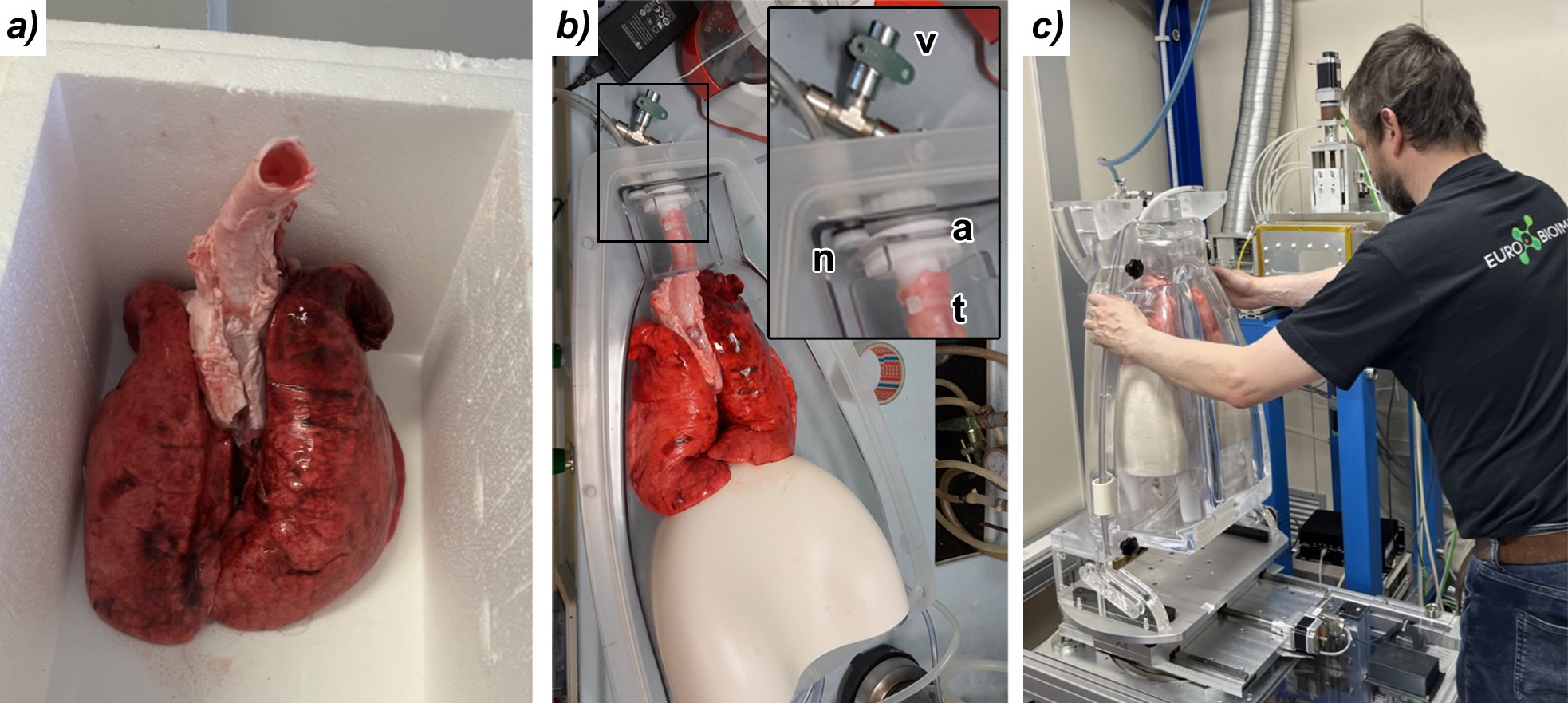


**Fig. S3 Anthropomorphic chest phantom design and mounting of the lung specimen. (a)** Defrosted pig lung prior to mounting. Excess connective tissue has been removed and the trachea trimmed to appropriate length. **(b)** Lung positioned in half of the inner shell of the phantom. Inset shows the drilling (n) used to apply negative pressure. A three-way valve (v) allows regulation of suction from the vacuum pump. The trachea (t) is secured with a cable strap to a pipe (a) connected to the surrounding air. **(c)** Placement of the lung-equipped inner shell into the outer radiation- absorbing shell, mounted on the synchrotron imaging stage.
